# Evolution of Functional Diversity Among Actin-Binding *Profilin* Genes in Land Plants

**DOI:** 10.3389/fcell.2020.588689

**Published:** 2020-12-16

**Authors:** Dhananjay K. Pandey, Bhupendra Chaudhary

**Affiliations:** School of Biotechnology, Gautam Buddha University, Greater Noida, India

**Keywords:** profilin, actin, evolution, expression, genomics, plants

## Introduction

The profilins (PRFs) are low-molecular weight, cytosolic proteins made up of 129–133 amino acids and tightly control cell-cytoskeleton architecture mainly through actin polymerization (Christensen et al., 1996; Pollard and Cooper, 2009). The PRFs are the actin-binding proteins having conserved profilin-actin interacting regions (PAINRs) which are indispensable to the mechanism of actin-polymerization or -depolymerization process (Giehl et al., 1994; Pandey and Chaudhary, 2017). Globally, the size and location of PAINRs vary from 10 to 11 amino acids and between 57 and 128 amino acids in PRFs, respectively. Evidently, a hypervariable region at the C-terminal of PRF is responsible for their complex binding with actin filaments and regulates diverse functions (Sohn and Goldschmidt-Clermont, 1994). Although PRF proteins are present in almost all life forms including lower and higher plants, animals, fungi, protists and viruses, little is explicit about the PRF-mediated molecular interplay and the corresponding functional networks in land plants.

The *PRF* genes are ancient, evolutionarily conserved and functionally divergent among life kingdoms. Apparently, these genes are responsible for the maintenance of cell-walls by actin sequestration, nucleation and cytokinesis in both prokarya and eukarya (Magdolen et al., 1988). The advent of genomics and biotechnology has emphasized the emerging roles of *PRF* genes during plant growth and development that reveals their “*novel*” functional capabilities beyond their traditional contributions to the cytoskeleton maintenance. Their functional attributes in the root elongation, leaf morphology, epidermal expansion, flowering time phenotypes and seed germination have recently been explored in various plant species (Ramachandran et al., 2000; Müssar et al., 2015; Pandey and Chaudhary, 2016). Constitutive overexpression and silencing of *PRF* genes show strong effects on fiber initiation and elongation in cotton as well as flowering time phenotypes, stress tolerance and regulation of *in vitro* organogenesis in tobacco. Previously, we have characterized the molecular interactions of PRFs with proline-rich ligands including Arp2/3 complex, ARP4, ARP6 proteins, cell signaling polyphosphoinositides, and other actin-binding proteins during plant growth and development (Supplementary Figure 1) (Pandey and Chaudhary, 2016). These findings decisively endorse the invaluable roles of PRF genes not only in the traditionally established cytoskeleton maintenance but also in the key aspects of plant development which can be further exploited for the crop improvement programs.

## Evolution of *PRF* Gene Family in Land Plants

Comparative *PRF* phylogenetic analyses of various life forms including lower and higher plants, animals, fungi, protists, and viruses emphasized their ancient origin. Most plant and animal species have several *PRF* genes on contrary to few such genes in the lower life forms. Interestingly, *PRF* genes are evolutionarily preserved and emerged prior to the expansion of descendant species. The non-synonymous to synonymous nucleotide substitution ratio (Ka/Ks) reveal that the purifying selection is primarily responsible for the evolutionary stability of *PRF* genes. In addition, the segmental and tandem duplication events are also prominent for their structural/functional divergences among lineages (Supplementary Figures 2, 3). Such structural variations in *PRF* genes are probably attributed to the variations in the exon-intron architecture of paralogous and orthologous sequences across species (Bao et al., 2011; Pandey and Chaudhary, 2016). Remarkably, evolutionary succession is largely achieved by the insertion and/or deletion in the exon/intron regions of *PRF* genes (Rogozin et al., 2003; Babenko et al., 2004). Results show that the evolution of exonic and intronic regions of *PRF* genes occurred essentially before the divergence of eudicot and monocot species in the Plantae kingdom (Pandey and Chaudhary, 2017) (Supplementary Figure 2). On the contrary to higher plants, lower plant species generally contain one long exon possibly due to intron/exon gain; or exon-fission and it reflects their evolutionary divergence from higher plants. Intron-exon structural organization of orthologs and paralogs provides clues to interpret the functional diversification of *PRF* gene family with their ancient evolutionary footprints (Pandey and Chaudhary, 2017). This illustrated a fascinating trend of greater sequence diversity among *PRF* genes with their evolutionary origin in the polyphyletic mode. Furthermore, viral *PRFs* had probably originated from their ancient relatives through horizontal gene transfer, and it suggests the prevalence of evolutionary conservation of *PRF* genes among living and non-living. Interestingly, the evolutionary testing of lineage-specific PRFs shows that these proteins are essential in organisms' development and evolved in Paleozoic (545 MYA), Mesozoic (252 MYA) and Cenozoic (66 MYA) life forms. Thus, PRFs are the notable outcome of the continuous evolution under natural selection (Supplementary Figure 3). The *PRF* genes are prevalent during the evolution of land plants e.g., bryophytes and gymnosperms (~425 and 385 MYA, respectively) (Pandey and Chaudhary, 2017). Bayesian phylogenetic algorithm shows that KYMVIQGE and VIRGKKG amino acid motifs are distinct among PRF-homologs and -orthologs, respectively, and categorized as evolutionary residues. The signatures of residual conservation show distinct and apparent classification patterns comprising three or five amino acids such as LAPTG, PGQCN, MSWQ, GDYL, YVD, AAI, and KKT (Pandey and Chaudhary, 2017).

Furthermore, PRFs play a vital function in the actin polymerization and depolymerization processes through binding to PAINRs on the protein surface (Giehl et al., 1994). The PAINRs are composed of alanines, isoleucines, glutamine, glutamic acid, lysine, glycine, threonine, proline, and arginine which allow PRFs to interact with actin residues. The *Arabidopsis PRF* sequence (Accession number-AAG10091) consists of 134 amino acids and 11 amino acids long PAINRs (AIQEKGTPGMR) present at 64, 78–79, 81, 89, 94, 114–116, 120, 124 amino acid positions (Pandey and Chaudhary, 2017). The evolutionary conjunction of PAINRs is identified during the functional evolution of lineage-specific PRFs (Pandey and Chaudhary, 2017). Lineage-specific PRFs consisting of at least 11 residues also show large heterogeneity in the PAINRs of plant, fungal, animal and cyanobacterial origin which confirms their ancient evolution in the primitive life forms (Pandey and Chaudhary, 2017).

## Emerging roles of *PRF* Genes During Plant Development and the Underlying Mechanisms

In response to endogenous or external signals, the cytoskeleton modifications in plants occur in an exceedingly coordinated manner preferably by the PRF-mediated polymerization of sequestered actin monomers and/or depolymerization of actin proteins (Pantaloni and Carlier, 1993). Despite striking discoveries into the genetics of cell-wall organization of plants (Taylor-Teeples et al., 2015), little is explicit about the PRF-mediated molecular interplay and the corresponding gene expression networks in plants. Several noteworthy examples highlighting the novel functions of *PRF* genes in plants are summarized here:

### Cotton Fiber Development

Cotton is one of the most important sources of natural fiber and remarkable result of single epidermal cell extension on the surface of ovules. Modern long and spinnable fibers are the extraordinary product of the evolutionary selection forces such as genomic polyploidy imposed on wild short-fuzz phenotypes during cotton evolution, followed by the millennium of selection under domestication. The comparative transcript profiling of the fiber cells of domesticated diploid and allopolyploid cotton species at three developmental stages with their wild counterparts reveal the expression up-regulation of cell-wall associated *PRF* genes (Chaudhary et al., 2008, 2009). In cotton, the *PRF* gene family is structured in its coding and flanking regions as a multigene family consisting of six members. These genes are up-regulated up to ~400 times in the elongating fiber cells of domesticated accessions compared to their wild forms (Bao et al., 2011).

Five *PRF* genes of the diploid cotton species express at high levels in the elongating fiber cells at 10 days post-anthesis (dpa) compared to the vegetative and floral tissues (Pandey and Chaudhary, 2019). *GhPRF1* gene expression at the 10 dpa fiber elongation stage is 9- and 16-fold higher than 5 and 20 dpa fiber tissues, respectively. Higher *PRF* expression at the fiber elongation stage is primarily important for cell extension and downstream cell signaling which declines very sharply at the maturation stage. Correspondingly, the extent of F-actin is also upregulated with increased filament length in the elongating fibers suggesting that temporal *PRF* transcript levels and actin filament organization are in equilibrium during fiber elongation (Pandey and Chaudhary, 2019). The temporal expression evolution of *PRF* paralog/homolog during cotton domestication gives evolutionary impressions of the selection of highly divergent transcription abundance, especially in the fiber development (Pandey and Chaudhary, 2017).

### Regulation of Flowering Phenotypes

**Figure 1:**
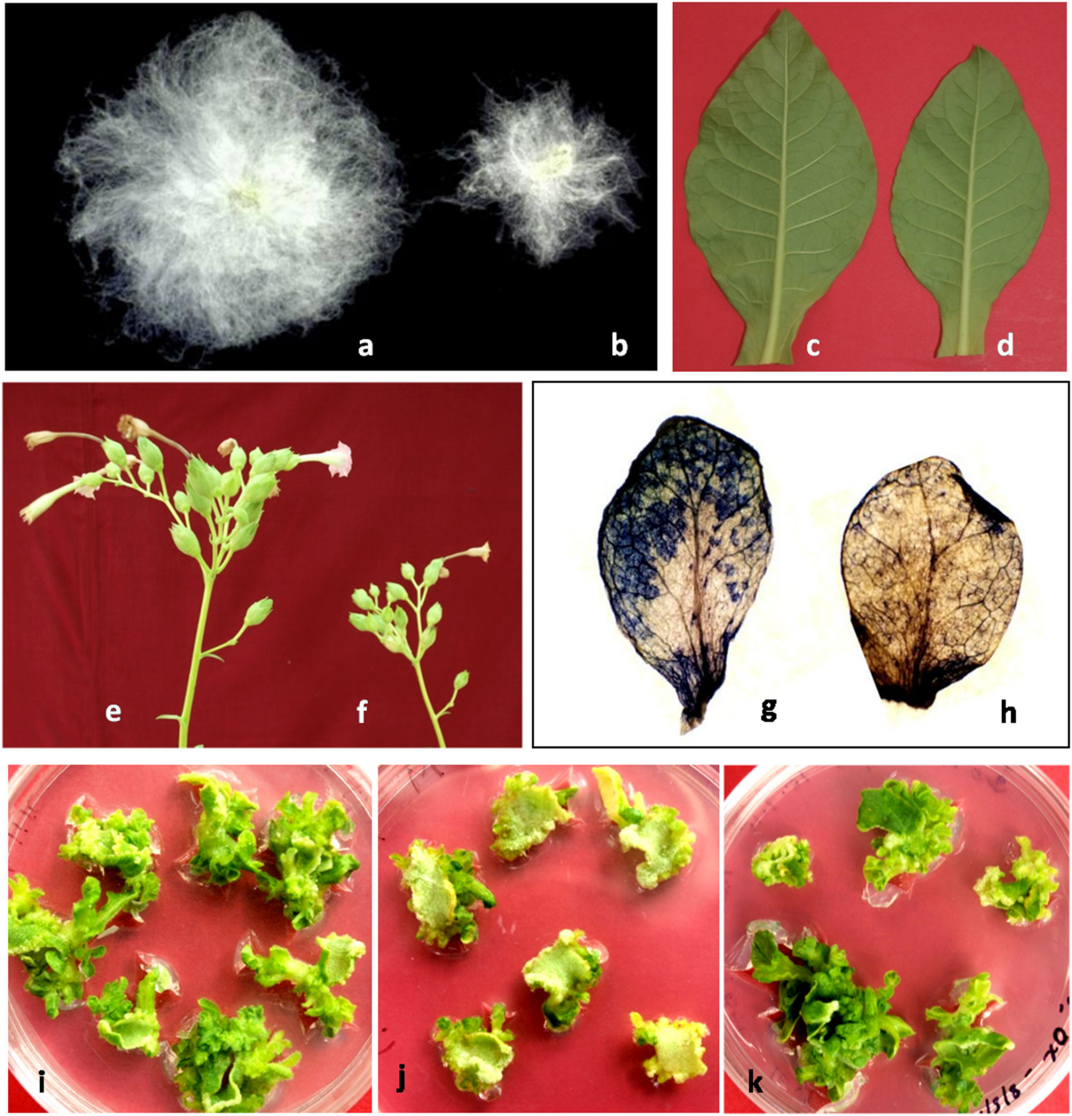
Functional diversity of actin-binding PRFs in plants. **(a)** domesticated cotton showing increased fibers length having ~400-fold increased *PRF* gene expression as compared to its wild counterpart **(b)** wild cotton fibers **(c)** increased size of leaf lamina in *PRF* overexpression line of tobacco **(d)** leaf lamina size of control plant **(e)** increased number of flowers in *PRF* overexpression line of tobacco **(f)** flowers in control plant **(g)** NBT-stained leaf of untransformed tobacco (grown on 50 mM NaCl concentration) showing higher ROS levels **(h)** NBT-stained leaf of *PRF* overexpression line of tobacco (grown on 50 mM NaCl concentration) **(i)** leaf explants of untransformed tobacco grown on *in vitro* culture medium **(j)** leaf explants of *gus*-transformed tobacco cultured on kanamycin antibiotic selection **(k)** leaf explants of *PRF* overexpression line of tobacco cultured on antibiotic selection (images in different panels are not to scale).

Previously, we show that the ectopic expression of *trans*-profilin (*trans*-*PRF*) gene in tobacco results in the hyperactivation of apical meristem, early flowering and a relative increase in the flower number per plant (Pandey and Chaudhary, 2016). The apical meristem tissues of *PRF* transgenics show coordinated expression of flowering-associated *FT4, SOC1, FLC1*, and *FT1* genes and a positive flowering regulator *AP1* gene. Moreover, protein-protein interactions and expression profiling reveal that Actin-Related Protein 4 (*ARP4*) and *ARP6* genes are upregulated in the vegetative and floral tissues of *PRF* transgenics. These results establish a novel and systematic functional relationship between *trans*-*PRF* gene expression and early flower primordium initiation in *PRF* trasngenics (Supplementary Figure 1). The *PRF*-overexpression lines of tobacco also exhibit increased plant height, internode length, leaf size and flower number per inflorescence without yield penalties (Figure 1) (Pandey and Chaudhary, 2016). On the contrary, *PRF*-downregulation significantly reduces (~40%) the initiation of healthy floral buds, normal stamen and pollen production, and flower growth. Floral organ development is severely defected in *PRF* silencing lines with shortened staminal tube coverage and filament duration, reduced stamen number, indehiscence of anthers, abrupt and uncoordinated increase in the style duration, and irregular seed shape (Pandey and Chaudhary, 2016). Due to the dominance of aberrant floral phenotypes of *PRF*-silencing lines, the average number of flowers per branch significantly decreases compared to both *PRF*-overexpression lines and control tobacco plants (Pandey and Chaudhary, 2016). Besides the traditionally established role of PRFs in the cytoskeleton maintenance, these observations strongly recommend their indispensable roles in novel aspects of plant development. The development of enhanced agronomic traits in the *PRF* overexpression lines has enhanced our understanding of the novel and vital roles of *PRF* genes that could be utilized in their future deployment in crop improvement programs (Pandey and Chaudhary, 2016).

### Stress Tolerance

The cell-wall is the outermost protective layer of a plant cell involved in the cytoskeleton integrity and provides conditions for the cell growth, development and defense from external stress (Le Gall et al., 2015; Höfte and Voxeur, 2017). Evidently, mass-spectrometry data reveal that up-regulation of PRFs in the root tissues of *Cucumis* and barley species under the salt treatment conditions (Du et al., 2010; Fatehi et al., 2012). Moreover, *PRF* gene expression elevation in a succulent halophyte *Suaeda aegyptiaca* under salinity stress further confirmed the role of PRFs in stress tolerance (Askari et al., 2006). These data provide evidence that PRFs are one of the foremost cytoskeleton-associated proteins that play vital roles during abiotic stress tolerance in plants. We have also investigated the effect of salt stress (up to 50 mM NaCl concentration) on tobacco *PRF*-overexpression transgenic lines under greenhouse conditions. Remarkably, oxidative stress measurement of these lines shows higher salt tolerance compared with control plants. Apparently, in response to various environmental stress conditions, the increased levels of oxidative stress produce highly reactive chemical molecules known as reactive oxygen species (ROS). Increased accumulation of ROS may cause cellular damages mainly through the degradation of lipids, proteins and nucleic acids (Das and Roychoudhury, 2014). Therefore, the cellular ROS levels of *PRF*-overexpression lines grown under salt stress conditions are measured by nitro blue tetrazolium (NBT) staining of the target tissues (Grellet Bournonville and Díaz-Ricci, 2011). The leaf tissues of *PRF*-overexpression tobacco lines grown under salt stress conditions show relatively decreased levels of ROS and establish a novel role of *PRF* genes in stress management in plants (Figure 1).

### Enhanced Magnitude of *in vitro* Organogenesis

A prerequisite to the successful *in vitro* micropropagation and genetic modification of a crop species is the availability of an efficient, robust and reproducible regeneration system. *In vitro* regeneration process largely involves in the transcriptional reprogramming of soma-cells to enable cellular totipotency. We observe that overexpression of *trans*-*PRF* gene under a constitutive promoter enhances the number of *in vitro* shoot formation (up to 14%) in tobacco. The molecular basis of such enhanced emergence of shoot-initial on the edges of explants is directly linked to the relative transcription of CLAVATA1 (*CLV1*) and WUSCHEL (*WUS*) genes/*trans*-factors that alter proportionally with the magnitude of organogenesis in *PRF*-overexpression lines. The *CLV1* and *WUS* genes are involved in the activation of essential signal transduction pathways required for the activation and formation of shoot primordial during *in vitro* organogenesis (Supplementary Figure 1) (Pandey and Chaudhary, 2016). Therefore, *PRF* genes have a direct role in the organogenesis process *in vitro* which can further be exploited for the improvement of agronomic traits in many crop species.

## Author Contributions

DP and BC conceptualized the research and outlines of the article and discussed all results and wrote the manuscript. DP performed all lab experiments cited in the article. All authors contributed to the article and approved the submitted version.

## Conflict of Interest

The authors declare that the research was conducted in the absence of any commercial or financial relationships that could be construed as a potential conflict of interest.

## Supplementary Material

Supplementary Figure 1 Model of complex interactive network of molecular factors associated to the *PRF* functional diversity.

Supplementary Figure 2 Evolutionary analyses of full length PRF proteins of various species of plant, animals, fungi, algae, cyanobacteria, and viruses is shown using Bayesian algorithm. Evolutionary distances among PRF sequences are proportional to the branch length on the phylogenetic tree.

Supplementary Figure 3 Existence and natural selection of *PRF* genes at evolutionary scale.
