# Supplementary material for: Evolution of Functional Diversity Among Actin-Binding Profilin Genes in Land Plants
Source: Front Cell Dev Biol. 2020 Dec 16;8:588689. doi: 10.3389/fcell.2020.588689 (PMC7772347; doi:10.3389/fcell.2020.588689)
Supplement: Supplementary Figure 1 — Model of complex interactive network of molecular factors associated to the PRF functional diversity. [file Presentation_1.PPT]

## Slide 1
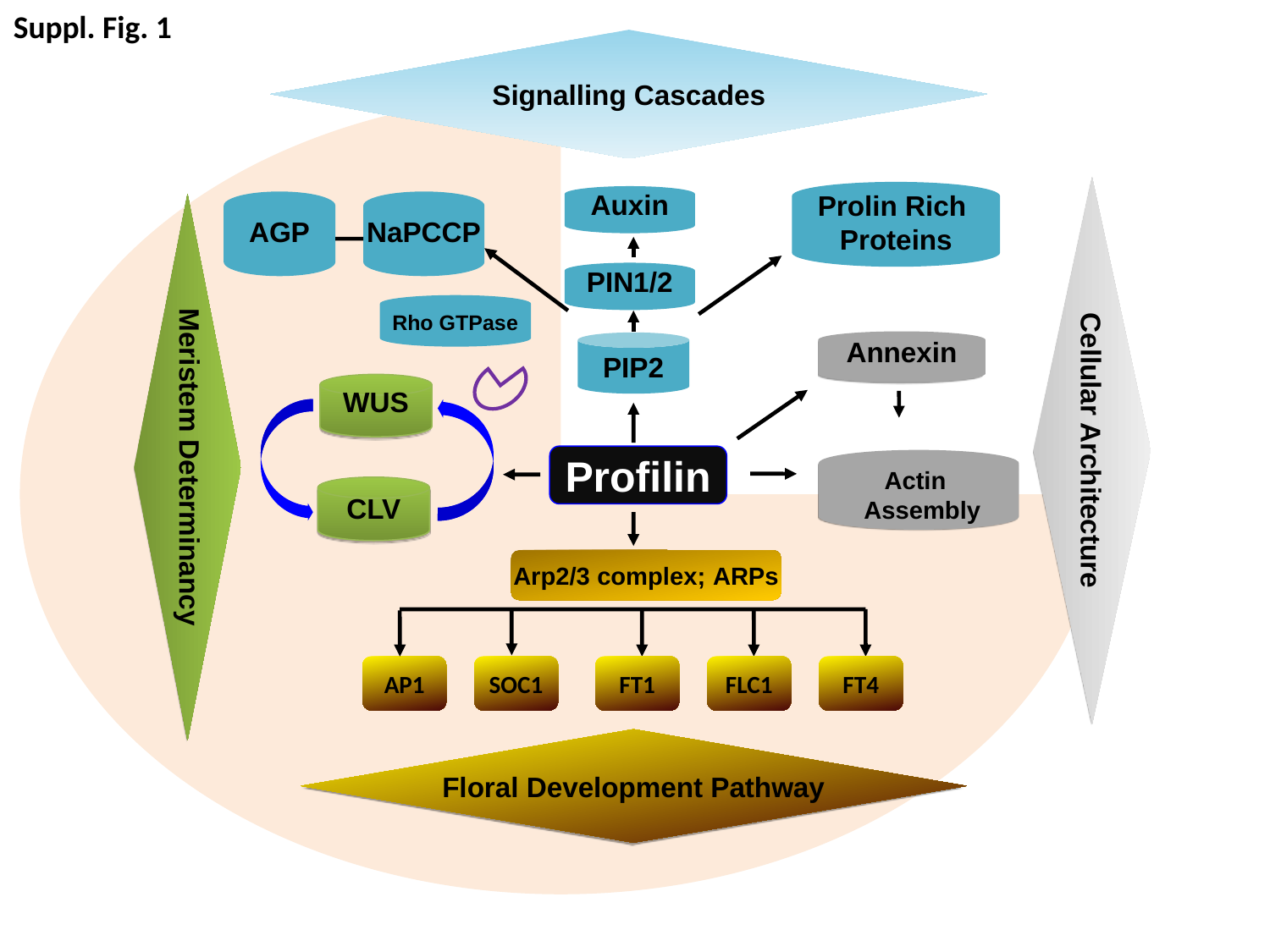

Suppl. Fig. 1
Signalling Cascades
Prolin Rich
Proteins
AGP
NaPCCP
Rho GTPase
PIP2
Cellular Architecture
Actin
 Assembly
WUS
Meristem Determinancy
CLV
Profilin
AP1
SOC1
FT1
FLC1
FT4
Floral Development Pathway
Annexin
Auxin
PIN1/2
Arp2/3 complex; ARPs
