# Supplementary figures and images for: Evolution of Functional Diversity Among Actin-Binding Profilin Genes in Land Plants
Source: Front Cell Dev Biol. 2020 Dec 16;8:588689. doi: 10.3389/fcell.2020.588689 (PMC7772347; doi:10.3389/fcell.2020.588689)

## Slide 1
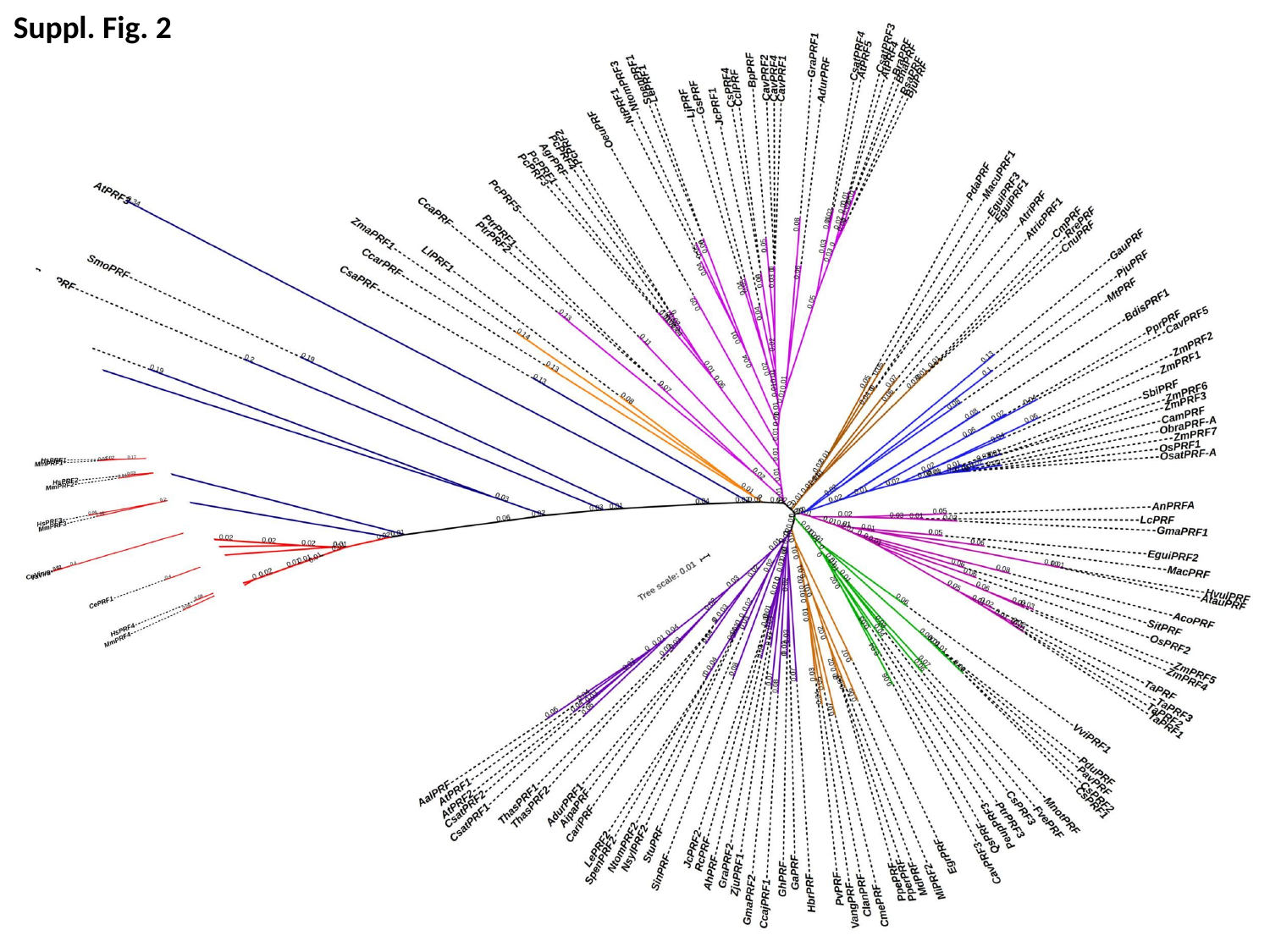

Suppl. Fig. 2

Supplement: Supplementary Figure 2 — Evolutionary analyses of full length PRF proteins of various species of plant, animals, fungi, algae, cyanobacteria, and viruses is shown using Bayesian algorithm. Evolutionary distances among PRF sequences are proportional to the branch length on the phylogenetic tree. [file Presentation_2.PPT]

## Slide 1
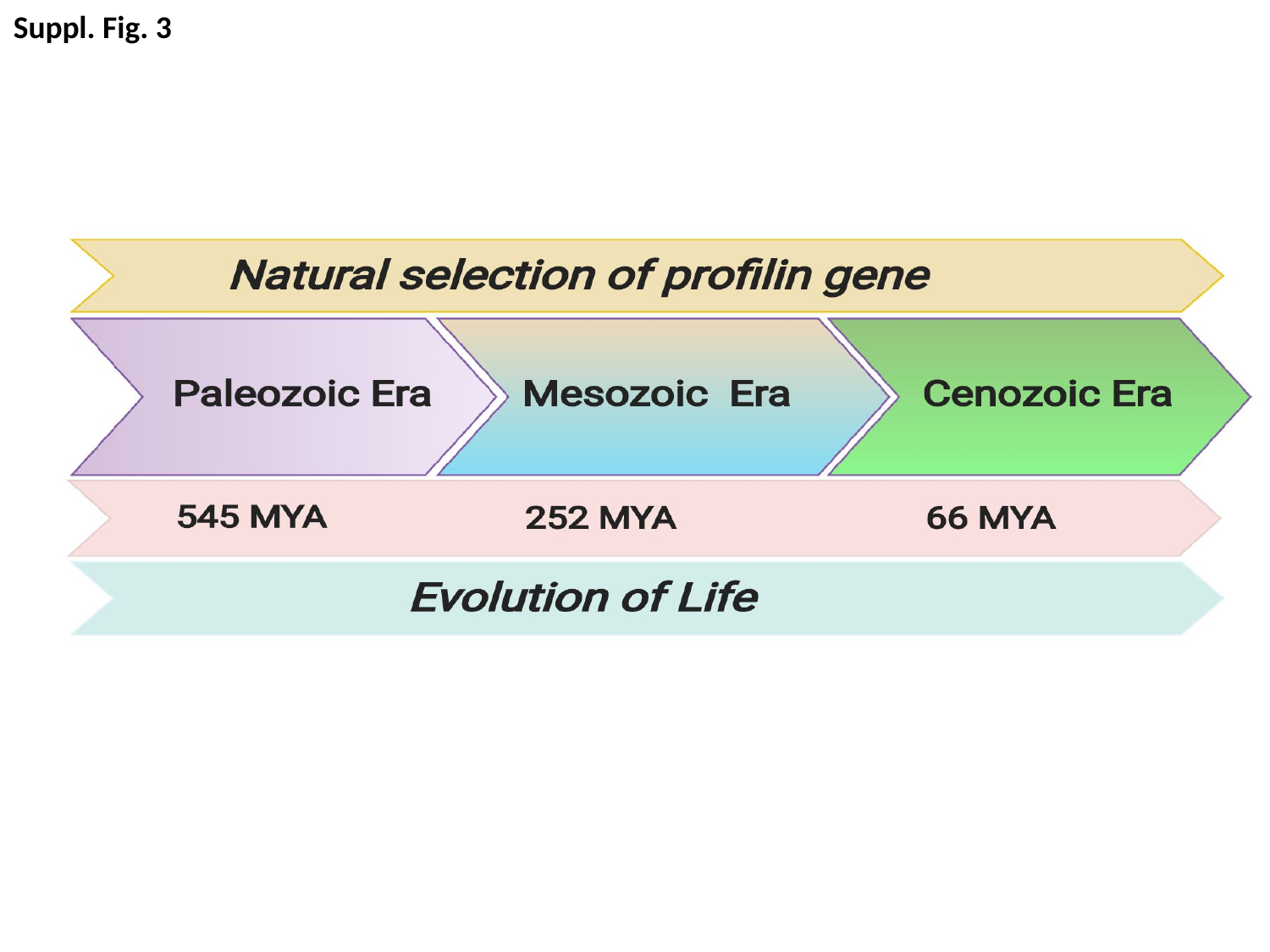

Suppl. Fig. 3

Supplement: Supplementary Figure 3 — Existence and natural selection of PRF genes at evolutionary scale. [file Presentation_3.PPT]
